# The efficacy and safety of core decompression for the treatment of femoral head necrosis: a systematic review and meta-analysis

**DOI:** 10.1186/s13018-019-1359-7

**Published:** 2019-09-11

**Authors:** Kun-chi Hua, Xiong-gang Yang, Jiang-tao Feng, Feng Wang, Li Yang, Hao Zhang, Yong-cheng Hu

**Affiliations:** 1 Department of Orthopedic Oncology, Tianjin Hospital, Tianjin, 300211 China; 2 Tianjin Medical University, Tianjin, 300070 China

**Keywords:** Core decompression, Femoral head necrosis, Meta-analysis

## Abstract

**Background:**

Core decompression (CD) is an important method for the treatment of osteonecrosis of the femoral head (ONFH). Few articles investigate the influence of core decompression on outcomes of ONFH. This study was carried out to observe the safety and effectiveness of core decompression in the treatment of ONFH.

**Methods:**

A comprehensive literature search of databases including PubMed, Embase, and Cochrane Library was performed to collect the related studies. The medical subject headings used were “femur head necrosis” and “Core decompression.” The relevant words in title or abstract included but not limited to “Osteonecrosis of the Femoral Head,” “femoral head necrosis,” “avascular necrosis of femoral head,” and “ischemic necrosis of femoral head.” The methodological index for nonrandomized studies was adopted for assessing the studies included in this review.

**Results:**

Thirty-two studies included 1865 patients (2441 hips). Twenty-one studies (1301 hips) using Ficat staging standard, 7 studies (338hips) using Association Research Circulation Osseous (ARCO) staging standard, and University of Pennsylvania system for staging avascular necrosis (UPSS) staging criteria for 4 studies (802 hips). All the studies recorded the treatment, 22 studies (1379 hips) were treated with core decompression (CD) alone, and 7 studies (565 hips) were treated with core decompression combined with autologous bone (CD Autologous bone). Nine subjects (497 hips) were treated with core decompression combined with autologous bone marrow (CD Marrow). Twenty-seven studies (2120 hips) documented the number of conversion to total hip replacement (THA), and 26 studies (1752hips) documented the number of radiographic progression (RP). Twenty-one studies recorded the types of complications and the number of cases, a total of 69 cases. The random-effect model was used for meta-analysis, and the results showed that the overall success rate was 65%. The rate of success showed significant difference on the outcomes of different stages. The rate of success, conversion to THA, and radiographic progression showed significant difference on the outcomes of ONFH using different treatments.

**Conclusions:**

Core decompression is an effective and safe method of treating ONFH. The combined use of autologous bone or bone marrow can increase the success rate. For advanced femoral head necrosis, the use of CD should be cautious. High-quality randomized controlled trials and prospective studies will be necessary to clarify the effects of different etiology factors, treatments, and postoperative rehabilitation. Until then, the surgeon can choose core decompression to treat ONFH depending on the patient’s condition.

**Level of evidence:**

I Meta-analysis

**Supplementary information:**

**Supplementary information** accompanies this paper at 10.1186/s13018-019-1359-7.

## Introduction

Osteonecrosis of the femoral head (ONFH) is interrupted or damaged by the blood supply to the femoral head, causing death and subsequent repair of bone cells and bone marrow components, which in turn leads to structural changes in the femoral head and collapse of the femoral head, causing joint pain and dysfunction in patients, and the disease is difficult to heal [1–3]. High disability rate is a common refractory disease in the field of orthopedics. There is a lack of effective treatment in clinical practice [4]. Most patients have to undergo total hip arthroplasty. The search for minimally invasive, safe, and effective treatment of femoral head necrosis has been a hot topic in orthopedic research [5]. Core decompression (CD) reduces the pressure in the bone, opens up the hardening zone that hinders the repair of osteonecrosis, stimulates the formation of blood vessels around the decompression tunnel, enhances the replacement of the new bone, and delays the progression of osteonecrosis [2–5]. A study confirms that CD combined with cytotherapy is a relatively good treatment for reducing the failure rate of early and mid-term ONFH patients [6]. Another study confirmed that CD combined with autologous bone marrow stem cells has achieved good results in early ONFH patients [7]. The purpose of this study was to summarize the efficacy of core decompression in the treatment of ONFH, to analyze the factors affecting the core decompression treatment of ONFH, and to evaluate the difference between the current core decompression and combined autologous bone or bone marrow therapy. Provide some useful advice for surgeons using core decompression therapy for ONFH.

## Materials and methods

### Literature search

PubMed-MEDLINE and Ovid-Embase were adopted for the comprehensive literature searches using a combination of the medical subject headings and relevant words in the title or abstract. The medical subject headings used were “core decompression” and “femur head necrosis.” The relevant words in the title or abstract included “femoral head necrosis,” “avascular necrosis of femoral head,” “ischemic necrosis of femoral head,” and “Osteonecrosis of the femoral head.” A cross-reference search was performed to identify additional studies. The search time is from January 1, 1980, to March 31, 2019, and the language is limited to Chinese and English. All retrieved records were added to an EndNote (Version X7; Thomson Reuters, New York, NY) library.

### Inclusion criteria

The inclusion criteria are as follows:

1. The published core decompression treatment of femoral head necrosis; the average follow-up time ≥ 1 year; the patient’s basic information (age at the time of treatment, gender, history of previous hip disease, etc.) record is complete; and if there is a control group, the two groups of patients are required to have no significant differences in basic information (age at the time of treatment, gender, history of previous hip disease, etc.).
2. Patients with femoral head necrosis were diagnosed by clinical physical examination and imaging and required to be treated with core decompression.
3. All of the studies are core decompression treatment of femoral head necrosis, and patients undergo treatment to promote healing of the surgical site.
4. The main indicators of each study were number of successful operations, radiographic progression, complications, and secondary operations.

### Exclusion criteria

The exclusion criteria are as follows:

1. The studies that were not associated with core decompression for femoral head necrosis
2. The number of successful hips after core decompression therapy was not clearly recorded, or a clear definition of surgical success was not given.
3. No analysis of prognostic factors
4. Review, case report, meetings abstracts, animal studies, editorial letters, guidance or comments, etc.
5. Repeated studies
6. Lower literature quality scores

### Quality assessment

The case series study used the National Institute for Clinical Excellence (NICE) case series scoring standard for quality evaluation. The case-control study used the Newcastle-Ottawa Scale (NOS) for quality evaluation. The scale was divided into three parts: “selection,” “comparability,” and “exposure.” The random control trial (RCT) used a modified Jadad scale for quality assessment, including randomized mass, grouped concealment, double-blind, and sample outcomes. Two researchers independently conducted a rigorous quality evaluation of the retrieved literature in accordance with the above inclusion and exclusion criteria. When there were different opinions, the discussion was conducted and a third researcher was invited to participate in the review.

### Data extraction

Relevant information, including first author, year of publication, study design, country of study, total number and age of ONFH patients, use of autologous bone or bone marrow during surgery, and preoperative and postoperative hip staging and criteria, was extracted. The clinical outcomes of our study included overall surgical success rate (definition of successful surgery: during follow-up, Harris hip score (HHS) ≥ 70, no further THA surgery required, no radiographic progression), rate of conversion to THA, rate of radiographic progression, rate of success in different stages, and complications such as fracture, surgical site pain, hematoma, deep vein thrombosis, and infection.

### Statistical analysis

Descriptive analysis of indicators that cannot be combined or that are not suitable for consolidation. In our study, we used the extracted raw data to calculate the rate of conversion to THA, the rate of radiological progression, the success rate of different treatment methods, and the success rate of different stages in each study. According to the sample size corresponding to each group rate, the corresponding standard deviation is calculated, and the standard deviation of the rate and rate is used as the effect amount for meta-analysis. *X*^2^ test was used for quantification of statistical inconsistency between studies, and *I*^2^ values showed the degree of heterogeneity. When significant heterogeneity (*P* ≤ 0.1 and *I*^2^ ≥ 50%) was detected, studies were combined using a random-effects model. When no significant heterogeneity (*P* > 0.1 and *I*^2^ < 50%) was detected, studies were combined using a fixed-effects model. The differences between subgroups were further tested, and a value of *P* less than 0.05 was considered statistically significant.

The *X*^2^ test was used to compare the success rate of Ficat I, II, and III stage, the success rate of different treatment methods, the rate of conversion to THA of different treatment methods, and the radiographic progression rate of different treatment methods. The test level *α* value was 0.05. The process was statistically analyzed using the SPSS 20.0 statistical software package.

## Results

### Literature search

**Fig. 1:**
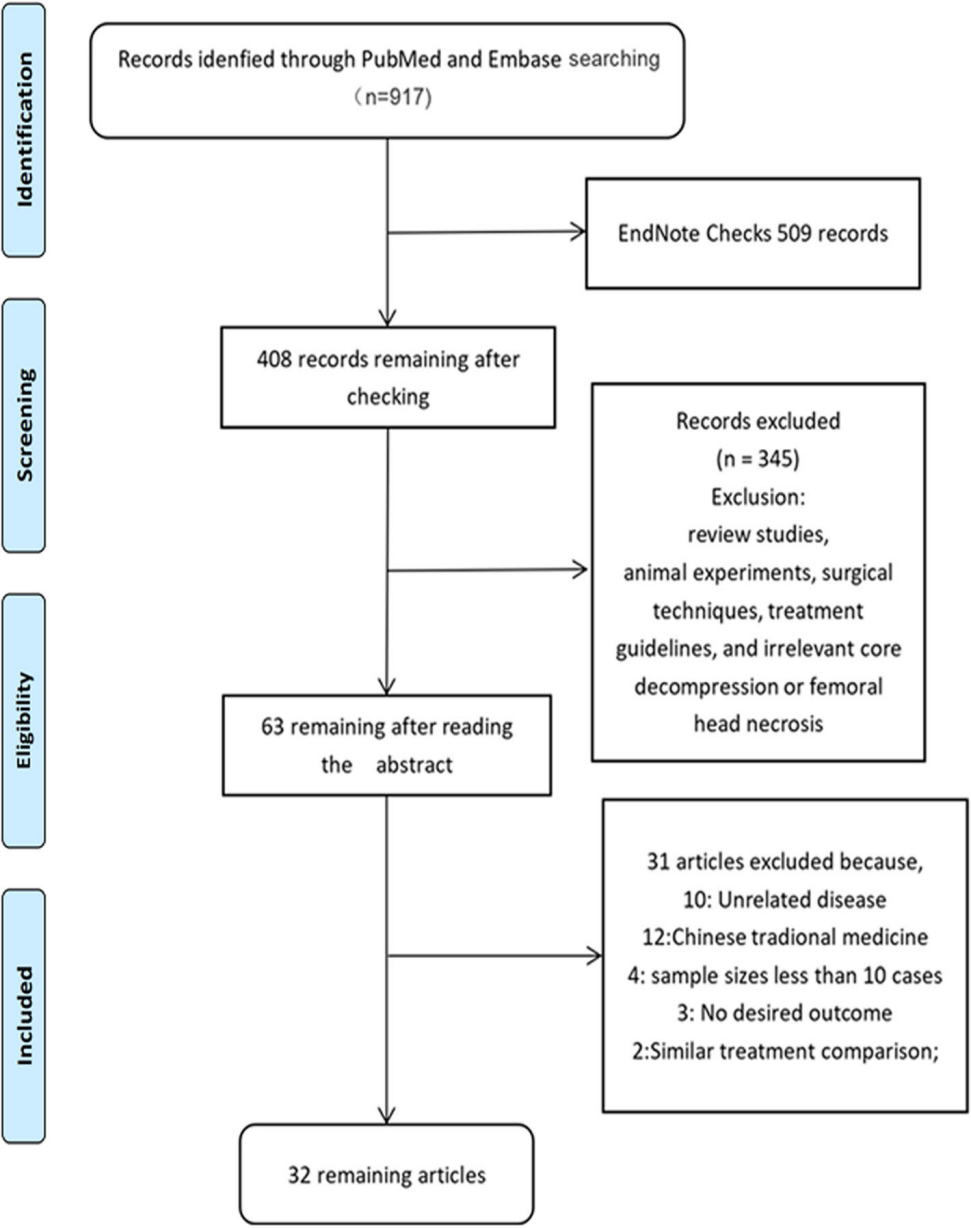
Flowchart of studies identification and selection

A flow diagram explaining the literature search strategy and study selection is shown in Fig. 1. A total of 917 articles were found by computer search, of which 384 were PubMed and 533 were retrieved from Embase (Additional file 1). Through the check processing of EndNote software, it was found that 509 articles were duplicated, and the remaining 408 articles of the title and abstract deletion of the literature were not in conformity with the remaining 63 articles after inclusion of the exclusion criteria. In order to further screen the full text of the reading, the remaining 32 studies were reproduced and the quality was completed. The evaluation included 3 RCTs [8–10], 26 case series studies [5, 11–35], and 3 case-control studies [36–38].

### Study characteristics

The basic characteristics of those studies are shown in Table 1. The study included 1865 patients (2441 hips) with an average age of approximately 37.72 (12–85) years, a female ratio of approximately 35.57%, and an average follow-up of 54.3 (2–228) months. The 32 studies included 3 RCTs, 26 case series studies, and 3 case-control studies. The RCT study used a modified version of the Jadad scale for quality evaluation, the average score of the three RCT studies was 5.67. The 26 case series used the NICE case series scoring criteria for quality evaluation, the average score of the study was 5.88. Three case-control studies used NOS scales for quality assessment with an average score of 8.67.

**Table 1:** Basic characteristics of included studies

Author	Year	Study type	Nation	Male/female	Age	Hips	Therapies	Evaluation standard	Study quality
Gangji	2011	RCT	Belgium	9/10	43.86	24	Control: CD Trial: CD+Marrow	Jadad	6
Zhao	2012	RCT	China	53/47	33.25	97	Control: CD Trial: CD+Marrow	Jadad	6
Pepke	2016	RCT	German	21/3	44.4	25	Control: CD Trial: CD+Marrow	Jadad	5
Zhang HJ	2010	Case-control	China	20/12	36.3	39	Control: CD Trial: CD+Marrow	NOS	8
Zhuo NQ	2012	Case-control	China	16/12	31.9	33	Control: CD Trial: CD+A.B	NOS	9
Guo HS	2018	Case-control	China	56/20	44.32	76	Control: CD Trial: CD+A.B	NOS	9
Warner	1987	Case series	USA	13/12	38	39	CD	NICE	6
Tooke	1988	Case series	USA	14/19	40	45	CD	NICE	6
Lausten	1990	Case series	Denmark	24/3	40	29	CD	NICE	7
Learmonth	1990	Case series	South Africa	21/11	37	41	CD	NICE	5
Fairbank	1994	Case series	USA	45/45	40	128	CD	NICE	6
Smith	1995	Case series	USA	59/33	41	114	CD	NICE	6
Markel	1996	Case series	USA	12/33	38.6	54	CD	NICE	6
Mont	1997	Case series	USA	22/28	34	79	CD	NICE	5
Powell	1997	Case series	USA	10/12	35	34	CD	NICE	6
Iorio	1998	Case series	USA	NA	40.8	33	CD	NICE	5
Bozic	1999	Case series	USA	21/13	38	54	CD	NICE	6
Steinberg	2001	Case series	USA	123/85	37	312	CD+A.B	NICE	5
Hernigou	2002	Case series	USA	75/41	31	189	CD+Marrow	NICE	6
Lieberman	2004	Case series	USA	6/9	47	17	CD	NICE	5
Belmar	2004	Case series	USA	NA	NA	302	CD	NICE	5
Song	2007	Case series	South Korea	120/16	36.1	163	CD	NICE	6
Li YP	2007	Case series	China	19/6	37.2	36	CD+A.B	NICE	5
Ji WF	2008	Case series	China	71/16	47	103	CD+Marrow	NICE	5
Xu WH	2009	Case series	China	26/14	35.6	42	CD+A.B	NICE	5
Wang	2010	Case series	China	36/9	37.5	59	CD+Marrow	NICE	5
Cao B	2010	Case series	China	37/18	39.8	61	CD+A.B	NICE	5
Yang J	2010	Case series	China	50/5	35	85	CD	NICE	5
Zhao Y	2011	Case series	China	16/6	Range (16–51)	25	CD	NICE	6
Chotivichit	2012	Case series	Thailand	3/7	36.18	11	CD+Marrow	NICE	6
Chotivichit	2014	Case series	Thailand	18/14	31.9	34	CD+Marrow	NICE	6
Chen XT	2015	Case series	China	31/19	36.2	58	CD+A.B	NICE	6

Note: *NA* not available, *CD* core decompression, *CD*+*Marrow* core decompression combined with autologous bone marrow, *CD*+*A*.*B* core decompression combined with autologous bone, *NICE* National Institute for Clinical Excellence, *NOS* Newcastle-Ottawa Scale

### Clinical outcomes

**Fig. 2:**
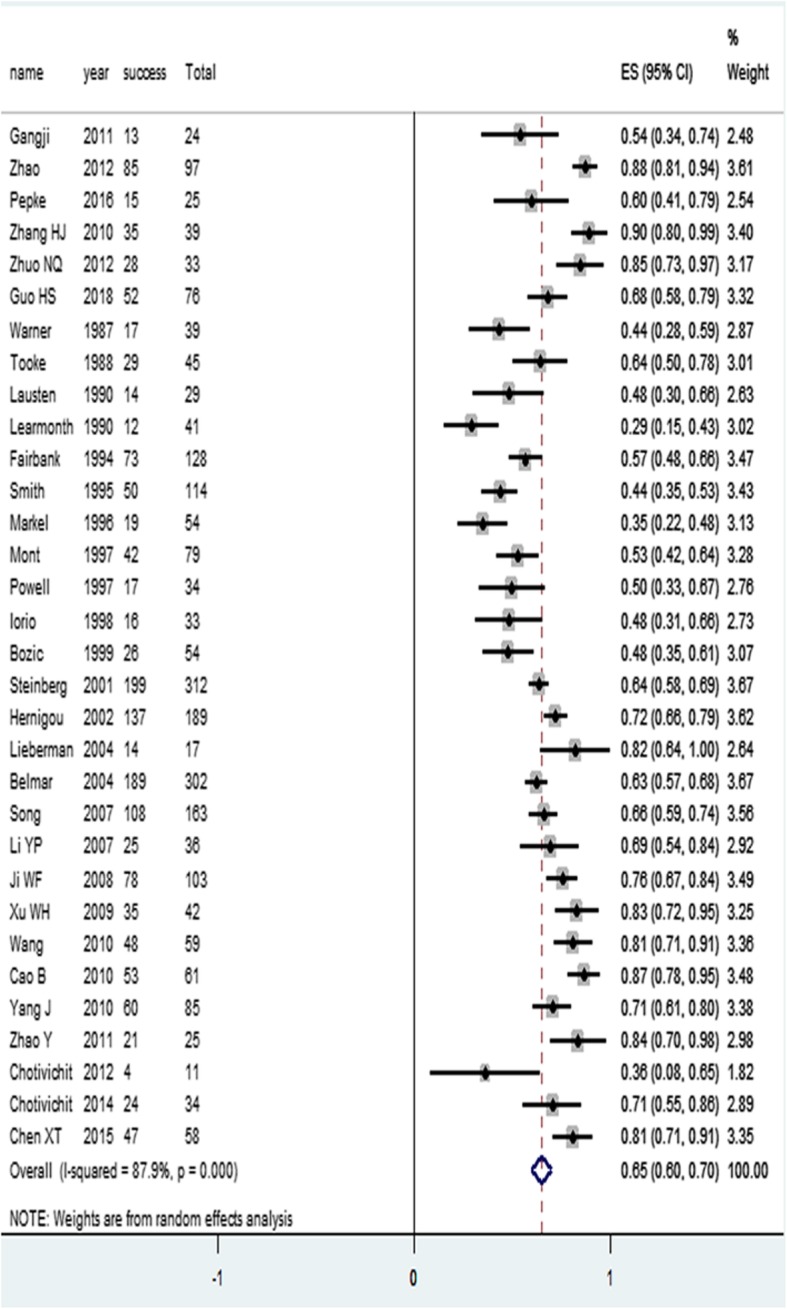
Overall success rate meta-analysis

Clinical outcomes of included studies are shown in Table 2. Meta-analysis using a random-effects model due to heterogeneity showed an overall effect size (ES) of 0.65 (95% CI (0.60, 0.70)) (Fig. 2), as shown in the published core decompression treatment of the femoral head necrosis. In the literature, the overall success rate after surgery was 65%.

**Table 2:** Clinical outcomes of included studies

Author	Year	Therapies	Hips	Hips to THA	Hips with RP	Staging methods	Complications	Follow-up (month)
Gangji	2011	Control: CD Trial: CD+Marrow	Control: 11 Trial: 13	Control: 3 Trial: 2	Control: 8 Trial: 3	ARCO	Pain: 4 Infection: 1	60
Zhao	2012	Control: CD Trial: CD+Marrow	Control: 44 Trial: 53	Control: 5 Trial: 2	Control: 10 Trial: 2	ARCO	None	60
Pepke	2016	Control: CD Trial: CD+Marrow	Control: 14 Trial: 11	Control: 6 Trial: 4	Control: 6 Trial: 4	ARCO	NA	24
Zhang HJ	2010	Control: CD Trial: CD+Marrow	Control: 15 Trial: 24	NA	Control: 2 Trial: 1	ARCO	None	18
Zhuo NQ	2012	Control: CD Trial: CD+A.B	Control: 12 Trial: 21	Control: 4 Trial: 1	Control: 4 Trial: 1	Ficat	None	30
Guo HS	2018	Control: CD Trial: CD+A.B	Control: 41 Trial: 35	NA	Control: 6 Trial: 1	Ficat	Pain: 1	30
Warner	1987	CD	39	19	23	Ficat	Fracture: 1 Hematoma: 1	16
Tooke	1988	CD	45	16	16	Ficat	Fracture: 1	36
Lausten	1990	CD	29	15	11	Ficat	Pain: 1	17.2
Learmonth	1990	CD	41	18	34	Ficat	Fracture: 1	32
Fairbank	1994	CD	128	55	81	Ficat	Fracture: 4 Perforation of femoral head: 1 Deep venous thrombosis: 2 Retained drain: 1	132
Smith	1995	CD	114	64	81	Ficat	Infection: 3 Fracture: 2 Hematoma: 2 Non-fatal pulmonary embolism: 1 Deep venous thrombosis: 1 Reflex sympathetic dystrophy: 1	40
Markel	1996	CD	54	26	NA	Ficat	Fracture: 2 Infection: 1	27.1
Mont	1997	CD	79	37	37	Ficat	NA	144
Powell	1997	CD	34	6	9	Ficat	None	48
Iorio	1998	CD	33	11	17	Ficat	NA	60
Bozic	1999	CD	54	28	34	Ficat	Fracture: 1 Hematoma: 1	97
Steinberg	2001	CD+A.B	312	113	113	UPSS	NA	48
Hernigou	2002	CD+Marrow	189	34	52	Ficat	Pain: 1 Pneumonia: 1 Alloimmunization: 1	84
Lieberman	2004	CD	17	3	3	Ficat	Pain: 4	53
Belmar	2004	CD	302	113	NA	UPSS	NA	46
Song	2007	CD	163	50	NA	Ficat	Heterotopic ossifications: 23 Fracture: 1	87
Li YP	2007	CD+A.B	36	4	4	ARCO	NA	23.4
Ji WF	2008	CD+Marrow	103	NA	NA	UPSS	None	26
Xu WH	2009	CD+A.B	42	NA	NA	Ficat	None	38
Wang	2010	CD+Marrow	59	7	14	ARCO	NA	27.6
Cao B	2010	CD+A.B	61	NA	22	Ficat	NA	26.4
Yang J	2010	CD	85	6	19	UPSS	None	57.6
Zhao Y	2011	CD	25	2	NA	Ficat	NA	73
Chotivichit	2012	CD+Marrow	11	2	8	Ficat	NA	42.6
Chotivichit	2014	CD+Marrow	34	10	9	Ficat	Pain: 4	25.8
Chen XT	2015	CD+A.B	58	11	11	ARCO	NA	34.05

Note: *NA* not available, *CD* core decompression, *CD*+*Marrow* core decompression combined with autologous bone marrow, *CD*+*A*.*B* core decompression combined with autologous bone, *THA* total hip replacement, *RP* radiographic progression

**Fig. 3:**
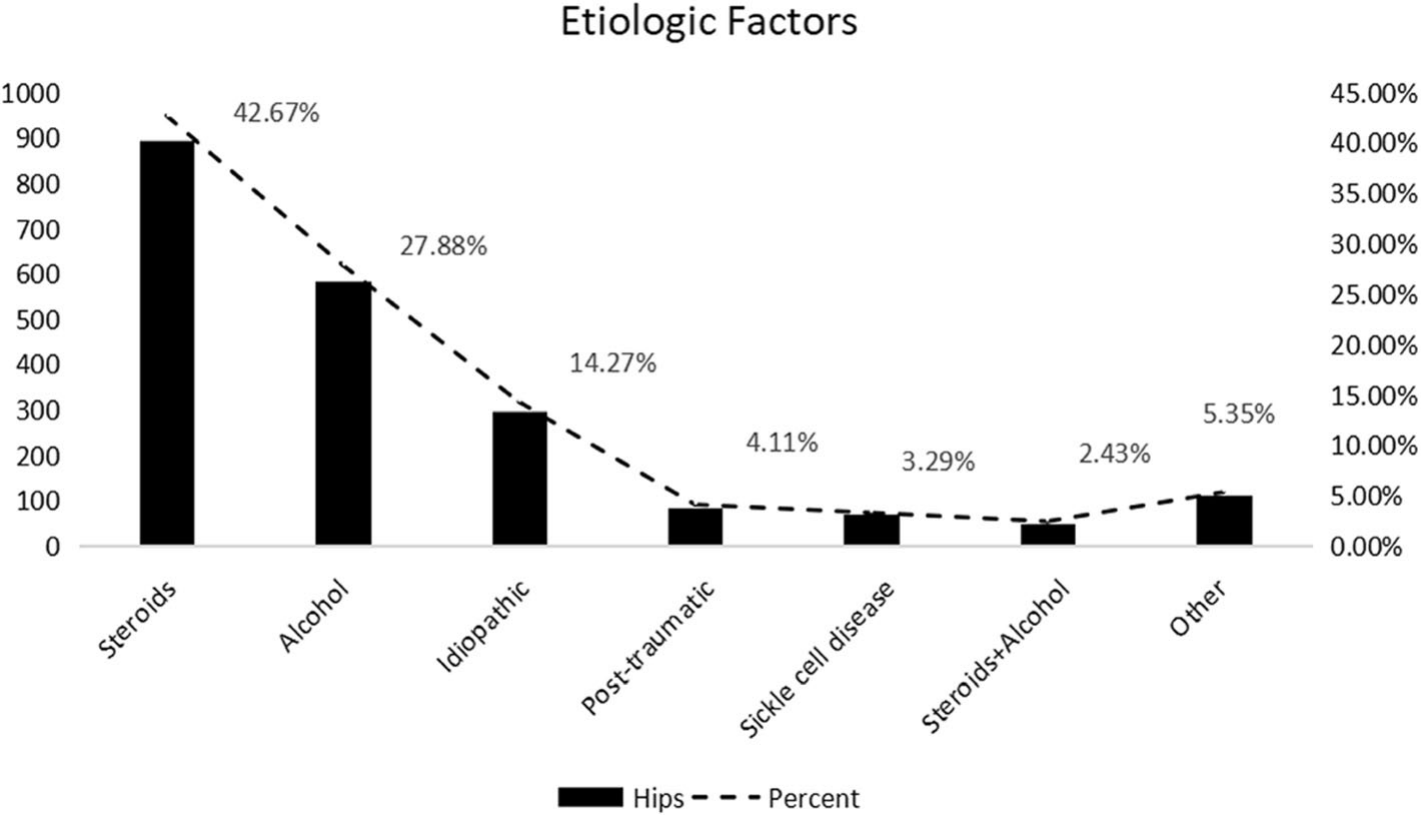
Etiologic factors

A total of 29 studies [5, 8, 10, 12–26, 28–38] (2095 hips) recorded the etiologic factors and the number of hips. The most common etiologic factor was steroids (894 hips, 42.7%), followed by alcohol (584 hips, 27.9%) and idiopathic (299 hips, 14.3%). The specific etiologic factors and number of hips are shown in Fig. 3.

A total of 21 studies [8, 10, 12, 14–16, 18–20, 22–26, 29, 30, 33, 34, 36–38] (1440 hips) recorded the type of complications and the number of cases, a total of 69 hips (4.79% 69/1440). Common complications are heterotopic ossifications (23 hips, 33.3%), pain (15 hips, 21.7%), and fracture (13 hips, 18.8%), and the specific complications and the number of hips are shown in Table 2.

We pooled analyses on the overall success rate; success rate of Ficat I, II, and III stage; the success rate of different treatment methods; the rate of conversion to THA of different treatment methods; and the radiographic progression rate of different treatment methods were conducted based on the data from the 32 studies.

### Success rate of different stages

**Fig. 4:**
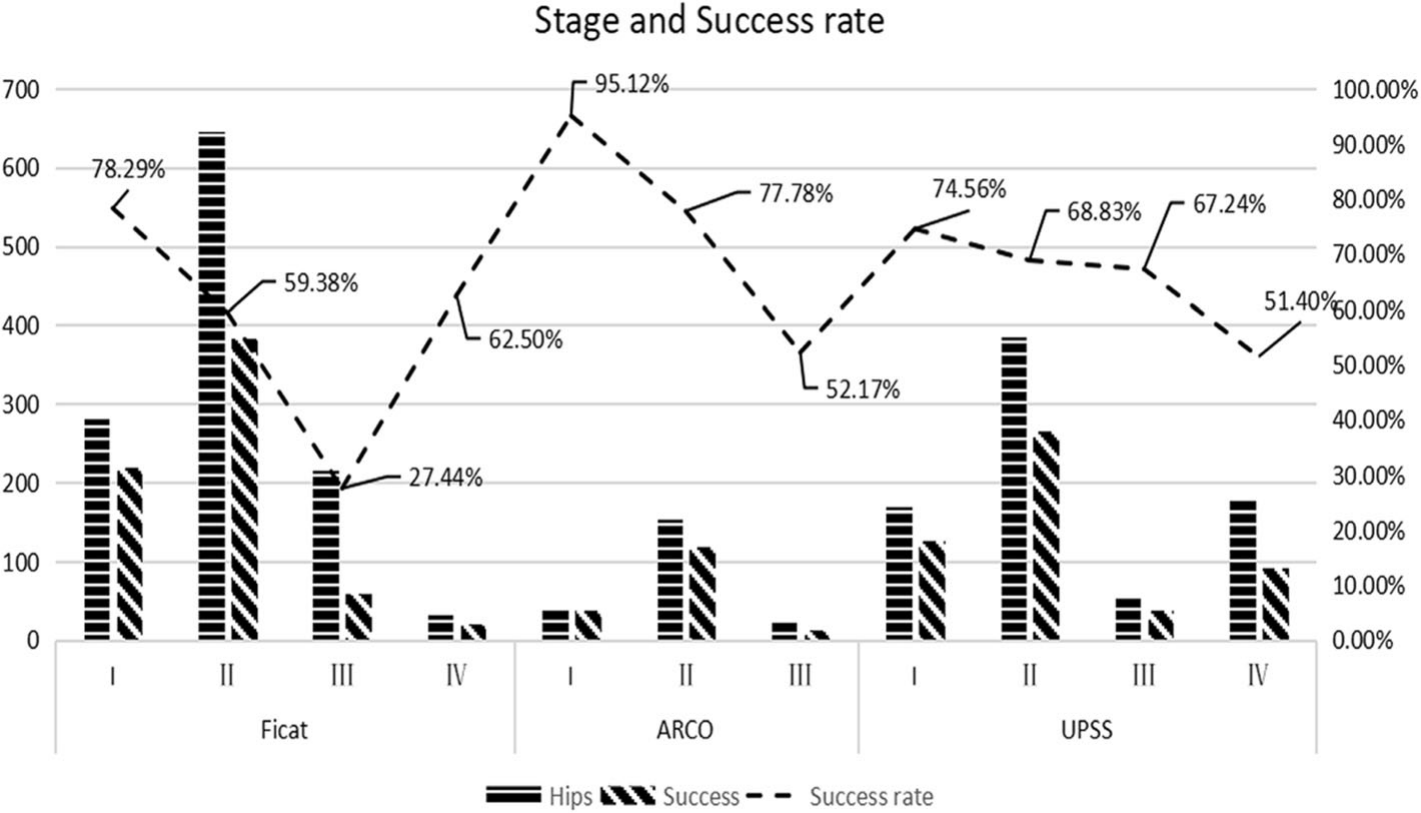
Stage and success rate

All studies recorded the staging of preoperative femoral head necrosis. Of these, 21 studies [12–25, 27, 30, 31, 33, 34, 36, 38] (1301 hips) used Ficat staging criteria, 17 studies [12, 13, 15–25, 30, 31, 34, 36] included stage I cases, 21 studies [12–25, 27, 30, 31, 33, 34, 36, 38] included stage II cases, 16 studies [12–16, 18, 20, 21, 23–25, 27, 30, 31, 33, 34] included stage III cases, and only one study included stage IV cases; 7 studies [8–10, 28, 32, 35, 37] (338 hips) used the Association Research Circulation Osseous (ARCO) staging criteria, 6 studies [8, 10, 28, 32, 35, 37] included stage I cases, and 7 [8–10, 28, 32, 35, 37] studies included stage II cases, and 2 studies [28, 35] included stage III cases; 4 studies [5, 11, 26, 29] (802 hips) used the University of Pennsylvania system for staging avascular necrosis (UPSS) staging criteria, 4 studies [5, 11, 26, 29] included stage I cases, 4 studies [5, 11, 26, 29] included stage II cases, 3 studies [5, 11, 26] included stage III cases, and 2 studies [5, 11] included stage IV cases (Fig. 4).

Considering the impact of the study and the number of cases on the outcome, this article only compares the success rates of Ficat I (220/280 78.29%), II (383/645 59.38%), and III (59/215 27.44%). Statistical analysis showed that the success rate of Ficat I (220/280 78.29%) > II (383/645 59.38%) > III (59/215 27.44%), the difference is statistically significant (*X*^2^ = 130.435, *P* < 0.05). Ficat I success rate and Ficat II success rate, the difference is statistically significant (*X*^2^ = 30.820, *P* < 0.05), Ficat II success rate and Ficat III success rate, the difference was statistically significant (*X*^2^ = 65.844, *P* < 0.05), Ficat I success rate and Fica III success rate, the difference is statistically significant (*X*^2^ = 127.980, *P* < 0.05).

### Success rate of different treatments

Treatment was documented in all studies, 22 studies [8–12, 15, 17–25, 29, 31, 33, 34, 36–38] (1379 hips) with core decompression (CD), and 5 studies [5, 27, 28, 36, 38] (565 hips) with core decompression and autologous bone (CD+A.B); 9 studies [8–10, 13, 14, 16, 26, 35, 37] (497 hips) used core decompression in combination with autologous bone marrow (CD+Marrow).

**Fig. 5:**
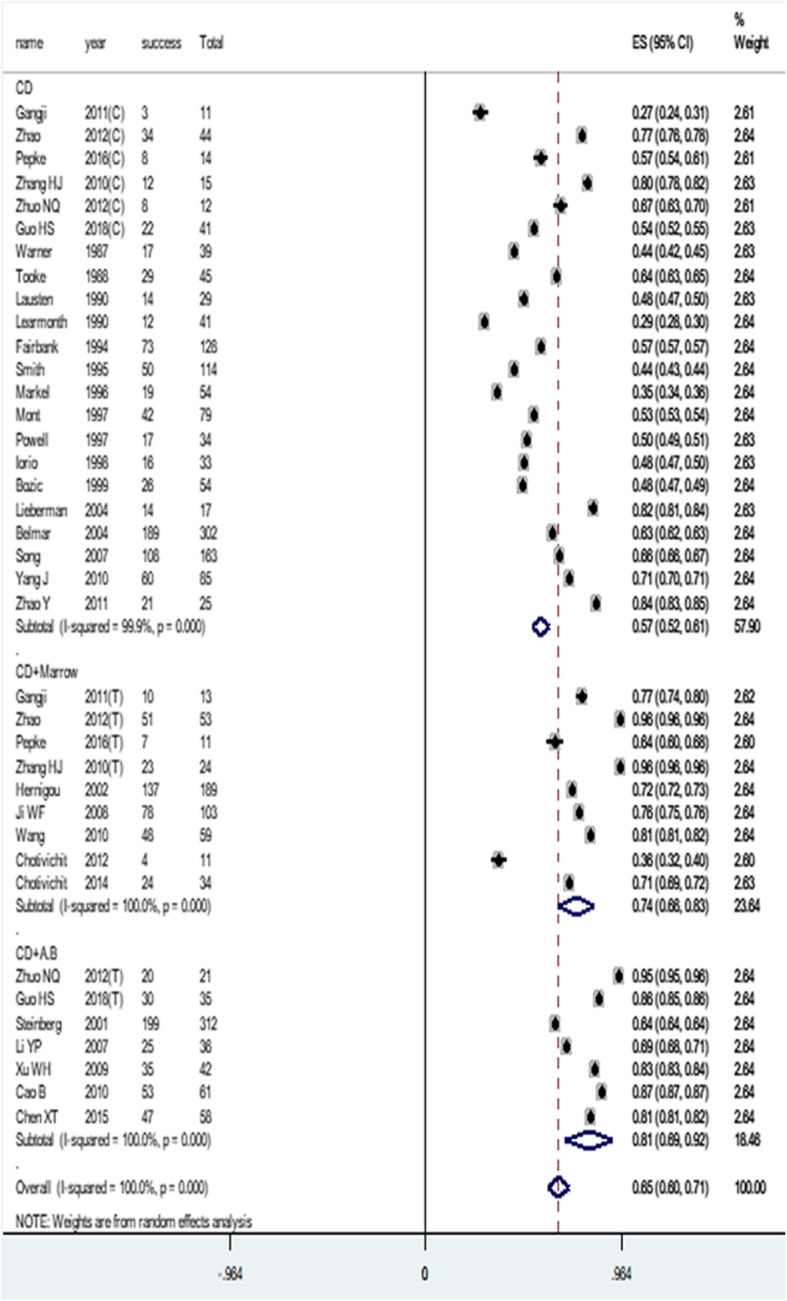
Meta-analysis of success rates for different treatments (C, control group; T, trial group)

Among the 32 studies using CD, CD+Marrow, and CD+A.B to treat ONFH, a total of 2441 hips were pooled into the meta-analysis on the success rate of different treatment methods, the control group and the experimental group of the case-control study and the RCT study were respectively considered as two groups of studies. The overall ES was 0.65 (95% CI, 0.60–0.71). In subgroup analysis, the ES and 95% CI were calculated as 0.57 (95% CI, 0.50–0.61), 0.74 (95% CI, 0.66–0.83), and 0.81 (95% CI, 0.69–0.92) in CD, CD+Marrow, and CD+A.B subgroup, respectively, as shown in Fig. 5. Moreover, the differences between the 3 subgroups were statistically significant (*P* < 0.05).

In order to clarify the differences between the three treatment methods, we performed an *X*^2^ test on the success rate. Statistical results display: success rate of CD and success rate of CD+Marrow, the difference is statistically significant (*X*^2^ = 53.236, *P* < 0.05); success rate of CD and success rate of CD+A.B, the difference was statistically significant (*X*^2^ = 36.245, *P* < 0.05); and success rate of CD+Marrow and success rate of CD+A.B, the difference is statistically significant (*X*^2^ = 2.067, *P* = 0.151).

### Rate of conversion to THA

**Fig. 6:**
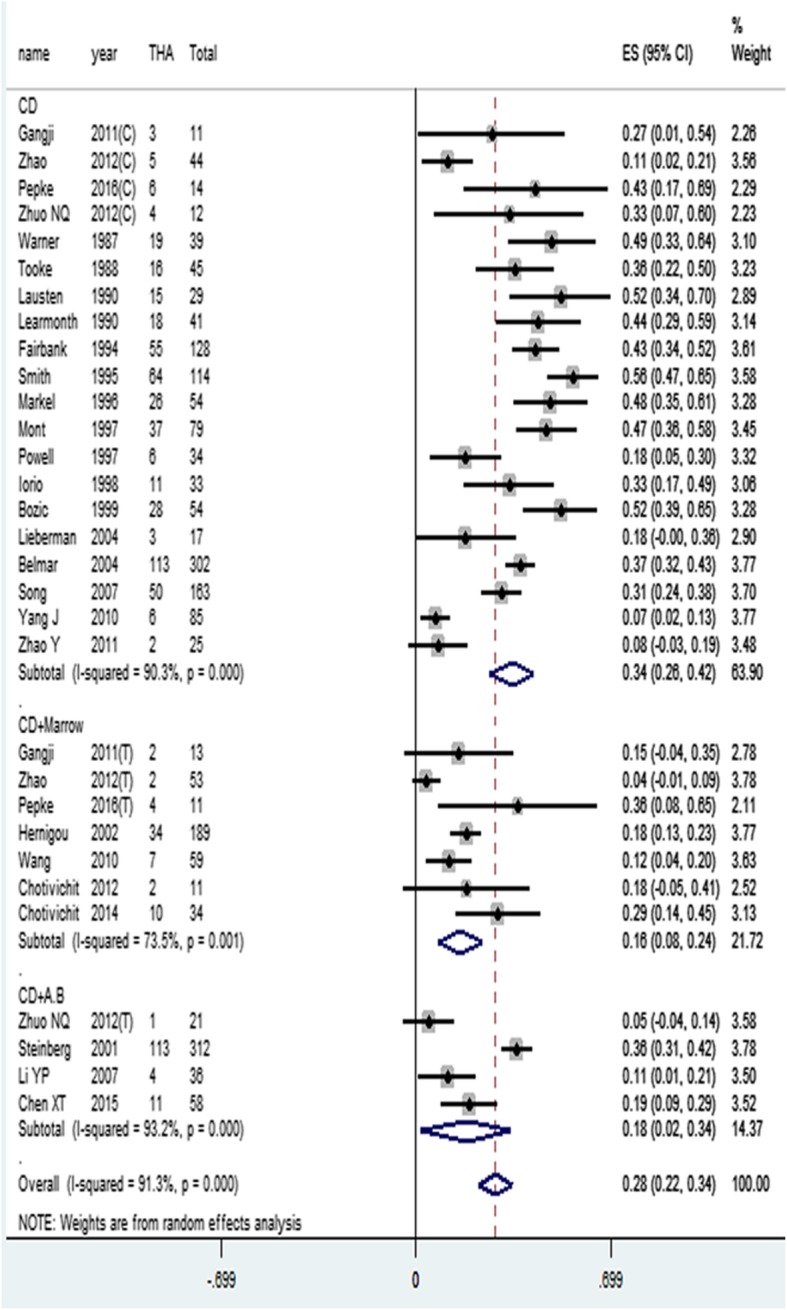
Conversion THA rate meta-analysis (C, control group; T, trial group)

Conversion to THA was recorded in 27 studies [5, 8–25, 28, 29, 31–36] using CD, CD+Marrow and CD+A.B for treatment of ONFH, a total of 2120 hips were pooled into the meta-analysis on rate of conversion to THA, the control group and the experimental group of the case-control study and the RCT study were respectively considered as two groups of studies. A conversion to THA was seen in 677 hips, which presented an overall ES of 0.28 (95% CI, 0.22–0.34). In subgroup analysis, the ES and 95% CI were calculated as 0.34 (95% CI, 0.26–0.42), 0.16 (95% CI, 0.08–0.24), and 0.18 (95% CI, 0.02–0.34) in CD, CD+Marrow, and CD+A.B subgroup, respectively, as shown in Fig. 6. Furthermore, the differences between the 3 subgroups were statistically significant (*P* < 0.05).

In order to clarify the differences between the three treatment methods, we performed an *X*^2^ test on the rate of conversion to THA. Statistical results display: CD and CD+Marrow rate of conversion to THA, the difference is statistically significant (*X*^2^ = 54.556, *P* < 0.05), CD and CD+A.B rate of conversion to THA, the difference is statistically significant (*X*^2^ = 6.614, *P* < 0.05), CD+Marrow and CD+A.B rate of conversion to THA, the difference is statistically significant (*X*^2^ = 20.565, *P* < 0.05).

### Rate of radiographic progression

**Fig. 7:**
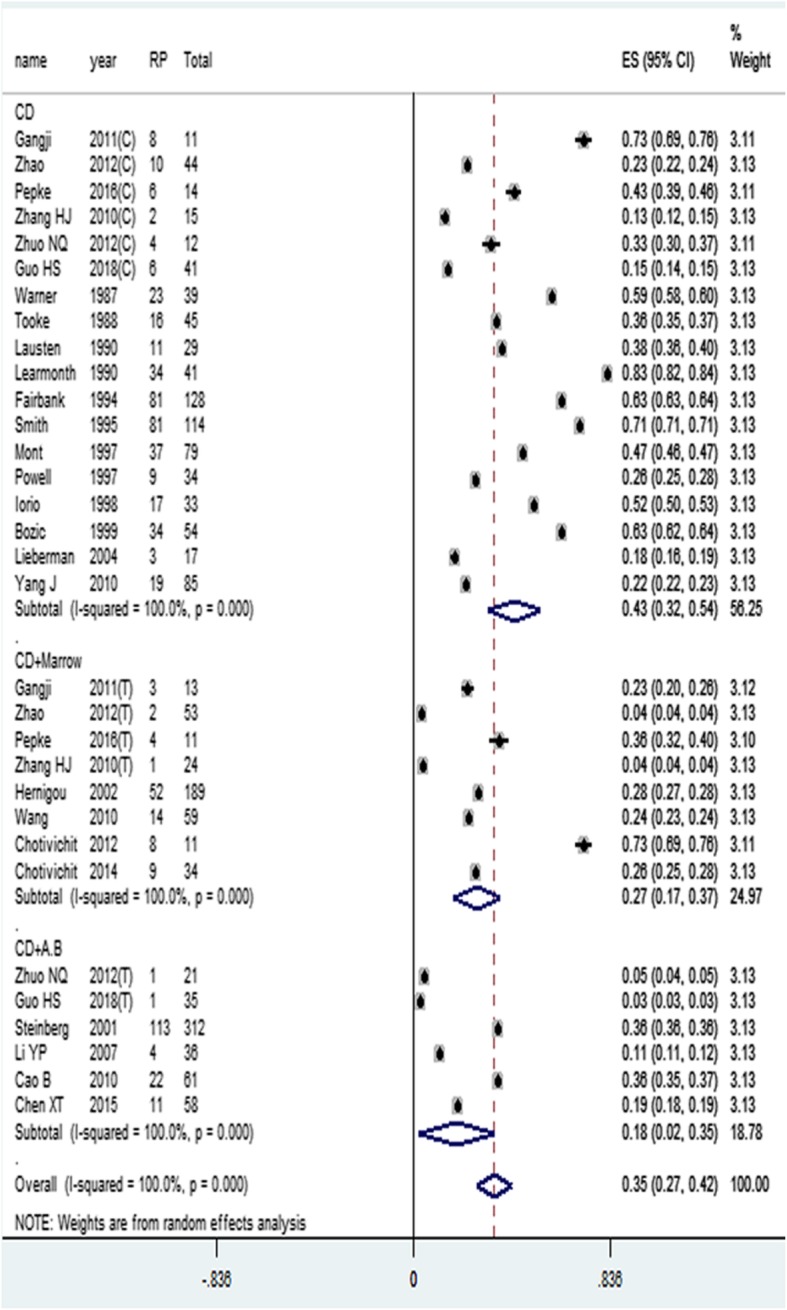
Rate of radiographic progression meta-analysis (C, control group; T, trial group)

Radiographic progression was recorded in 26 studies [5, 8–10, 12–19, 21–25, 27–29, 32, 33, 35–38] using CD, CD+Marrow and CD+A.B for treatment of ONFH, a total of 1752 hips were pooled into the meta-analysis on rate of radiographic progression, the control group and the experimental group of the case-control study and the RCT study were respectively considered as two groups of studies. A radiographic progression was seen in 646 hips, which presented an overall ES of 0.35 (95% CI, 0.27–0.42). In subgroup analysis, the ES and 95% CI were calculated as 0.43 (95% CI, 0.32–0.54), 0.27 (95% CI, 0.17–0.32), and 0.18 (95% CI, 0.02–0.35) in CD, CD+Marrow, and CD+A.B subgroup, respectively, as shown in Fig. 7. Furthermore, the differences between the 3 subgroups were statistically significant (*P* < 0.05).

In order to clarify the differences between the three treatment methods, we performed an *X*^2^ test on the rate of radiographic progression. Statistical results display CD and CD+Marrow rate of radiographic progression, the difference is statistically significant (*X*^2^ = 66.406, *P* < 0.05); CD and CD+A.B rate of radiographic progression, the difference was statistically significant (*X*^2^ = 47.894, *P* < 0.05); CD+Marrow and CD+A.B rate of radiographic progression, the difference is statistically significant (*X*^2^ = 3.420, *P* = 0.064).

## Discussion

The core decompression can be targeted to scrape the lesion to the peripheral wall of the bone, which is obviously oozing, that is, close to the normal bone tissue, effectively improving the blood circulation of the bone bed, facilitating the growth of the blood vessel along the tunnel into the femoral head and promoting the repair of the femoral head [39, 40]. Common surgical procedures include core decompression, core decompression plus autologous bone marrow and core decompression plus autologous bone grafting. This study has systematically reviewed the efficacy of core decompression therapy for ONFH. We analyzed the etiology factors, the type of complications and the number of hips, the influence of different preoperative staging on the prognosis, the success rate of different treatment methods, the rate of conversion to THA, and the rate of radiographic progression.

Twenty-nine studies (2095 hips) clearly documented the etiologic factors, mainly steroid and alcohol. However, each study was unable to provide the final treatment outcome for each of the etiologic factors, and we were unable to analyze the impact of the etiologic factors on the prognosis. Review literature found that alcohol and steroid are common causes of ONFH [41, 42], drinking alcohol and applying corticosteroids can cause lipid metabolism disorder [43, 44]. It leads to an increase in the volume of fat cells, fat embolism, etc. which plays an important role in the whole process of femoral head necrosis [45]. From an etiological perspective, it is an important measure to stay away from these risk factors while treating.

Complications were described in 21 studies (1440 hips). Due to the lack of information, there was no detailed statistical analysis of complications, only a rough calculation of the overall complication rate (4.79% 69/1440). Complications were described in 21 studies.

Of the 32 studies included, a total of three staging criteria were used, Ficat, ARCO, and UPSS, respectively. Considering the effect of the number of studies and the sample size on the results, only compare the success rates of the included Ficat I, II, and III cases. The success rate was I > II > III, and the difference was statistically significant. It is proved that preoperative staging is an important factor affecting postoperative, and the success rate of stage III is only 27.44%. The authors believe that CD should be considered carefully for cases of stage III. This procedure is especially suitable for young patients with early femoral head necrosis, for delaying or avoiding total hip replacement is of great significance [10, 19, 29, 32].

There are three treatment methods in the selected articles, CD, CD+Marrow, and CD+A.B. The three treatment methods have different success rates. Further statistical analysis shows that composite bone marrow or autologous bone can significantly improve the success rate. The success rate of CD+Marrow (74.0%) and the success rate of CD+A.B (81.0%) are both higher than the overall success rate (65.0%), and CD+A.B has the highest success rate. In addition, in the study using CD+A.B, the success rate of only one study [5] is lower than the overall success rate.

The authors noted that in addition to the above methods, there were reports of core decompression with vascular pedicle bone graft, core decompression combined with bone debridement, and core decompression combined with tantalum rod implantation, all of which achieved satisfactory clinical results [46, 47].

Twenty-seven studies (2120 hips) detail the conversion to THA under different treatments. Both CD+A.B and CD+Marrow significantly reduce the rate of conversion to THA compared to CD. Twenty-six studies (1752 hips) detail the radiographic progression under different treatments. Both methods significantly reduce the rate of radiographic progression compared to CD. These two results are also consistent with the success rate of three treatment methods.

Three studies [27, 33, 36] added additional bone morphogenetic proteins (BMP) during treatment, which caught our interest in this substance. Exogenous BMP has a positive effect on the treatment of femoral head necrosis [48]. BMP is widely present in the bone matrix, which induces osteogenic cells in normal bone tissue and generates bone and cartilage tissue in bone and surrounding soft tissues [49]. Zhuo et al. [36] believe that the addition of BMP can promote the repair of the femoral head, cure, or delay the progression of the disease.

Two studies [26, 30] used graft materials of allogeneic bone composite autologous bone during the treatment, which is also the current development trend of bone transplantation. Autologous bone provides the best osteogenic and osteoinductive capacity without immune rejection [50]. However, its defects are also very obvious, such as prolonged operation time, increased trauma area of the patient, insufficient bone supply and many complications in the donor site [51]. The use of allogeneic bone can avoid the defects of autologous bone, and its osteogenic and osteoinductive ability is also widely recognized. With the deepening of scientific research, the problem of immune rejection and disease transmission in allogeneic transplantation is also a very good solution [52, 53]. The prospect of application of allogeneic bone in the treatment of femoral head necrosis is worth looking forward to.

### Limitation

The current meta-analysis has certain limitations. First, there were only three RCTs in these studies, which led to a lower than expected level of evidence for our comprehensive comparison. Second, each study has different definitions of success, and assessing the diversity of standards will inevitably lead to bias in the final combined results. Third, some of the research lacks some important information, for example, etiology, complications, and postoperative rehabilitation of patients, this will lead to some factors that can only be used for simple calculation and analysis, and statistical tests cannot be performed. Finally, some innovative treatments are not included in the study, and the emergence of new methods requires a period of case accumulation in order to get a larger sample, which requires constant updating of the study in the future.

## Conclusion

Core decompression is an effective and safe method of treating ONFH. The combined use of autologous bone or bone marrow can increase the success rate. For advanced femoral head necrosis, the use of CD should be cautious. High-quality randomized controlled trials and prospective studies will be necessary to clarify the effects of different etiology factors, treatments, and postoperative rehabilitation. Until then, the surgeon can choose core decompression to treat ONFH depending on the patient’s condition.

## Supplementary information

**Additional file 1:** The searching strategies used in platforms of PubMed and EMBASE. (12.9 KB)

## Data Availability

The authors declare that all the data supporting the findings of this study are available within the article and its supplementary information files.

## Abbreviations

ARCO: Association Research Circulation Osseous
BMP: Bone morphogenetic proteins
CD Autologous bone: Core decompression combined with autologous bone
CD Marrow: Core decompression combined with autologous bone marrow
CD: Core decompression
ES: Effect size
HHS: Harris hip score
NICE: National Institute for Clinical Excellence
NOS: Newcastle-Ottawa Scale
ONFH: Osteonecrosis of the femoral head
RP: Radiographic progression
THA: Total hip replacement
UPSS: University of Pennsylvania system for staging avascular necrosis
