# Supplementary material for: The efficacy and safety of core decompression for the treatment of femoral head necrosis: a systematic review and meta-analysis
Source: J Orthop Surg Res. 2019 Sep 11;14:306. doi: 10.1186/s13018-019-1359-7 (PMC6737645; doi:10.1186/s13018-019-1359-7)
Supplement: Supplementary file 1 — Additional file 1: The searching strategies used in platforms of PubMed and EMBASE. (12.9 KB) [file 13018_2019_1359_MOESM1_ESM.docx]

**The searching strategies used in platforms of PubMed and EMBASE**

1. **PubMed:**

*#1 (Femur Head Necroses[MeTH]) OR (Aseptic Necrosis of Femur Head) OR (Ischemic Necrosis Of Femoral Head) OR (Avascular Necrosis of Femur Head) OR (Osteonecrosis of the femoral head)*

*#2 (Core decompression[MeSH]) OR ( Core decompression)*

*#3 #1 OR #2*

1. ***Embase:***

*#1 'femur head necrosis'/exp*

*#2* *femur AND head AND necrosis*

*#3 osteonecrosis AND of AND femoral AND head*

*#4 avascular AND necrosis AND of AND femur AND head*

*#5 ischemic AND necrosis AND of AND femoral AND head*

*#6 aseptic AND necrosis AND of AND femur AND head*

*#7 Osteonecrosis AND of AND the AND femoral AND head*

*#8 #1 AND #2 AND #3 AND #4 AND #5 AND #6 AND #7*

*#9 'core decompression'/exp*

*#10 core AND decompression*

*#11 #9 AND #10*

*#12 #8 AND #11*
